# *Lactobacillus paracasei* N1115 attenuates obesity in high‐fat diet‐induced obese mice

**DOI:** 10.1002/fsn3.3073

**Published:** 2022-10-20

**Authors:** Yanan Sun, Shanbin Chen, Fazheng Ren, Yixuan Li

**Affiliations:** ^1^ Key Laboratory of Precision Nutrition and Food Quality, Ministry of Education, Department of Nutrition and Health China Agricultural University Beijing China; ^2^ Key Laboratory of Functional Dairy, Ministry of Education, Department of Nutrition and Health China Agricultural University Beijing China

**Keywords:** gut microbiota, inflammation, *Lactobacillus paracasei* N1115, lipid metabolism, obesity

## Abstract

Disruption of the microbial structure of intestinal bacteria due to a high‐fat diet (HFD) is closely associated with metabolic disorders, such as obesity and type 2 diabetes. Probiotics are known to modulate the gut microbiota; therefore, we demonstrated the capability of *Lactobacillus paracasei* N1115 (*LC*‐N1115) to attenuate obesity. Four‐week‐old male C57BL/6J mice were fed a HFD for 12 weeks to induce obesity and were then randomized to supplemented placebo or *LC‐*N1115 treatment group for another 12 weeks. *LC*‐N1115 treatment reduced weight gain and liver fat accumulation as well as triglyceride, total cholesterol, and low‐density lipoprotein cholesterol levels. The administration of *LC*‐N1115 suppressed the expression of fatty acid synthase, interleukin‐1 β, and toll‐like receptor 4. Notably, the operational taxonomic units that negatively and positively correlated with the obesity phenotypes were enriched and reduced, respectively, in the *LC*‐N1115 treatment group. These results indicate that *LC*‐N1115 attenuates obesity by modulating the gut microbiota and the expression of lipid synthesis and proinflammatory cytokine genes.

## Graphical abstract

*Lactobacillus paracasei* N1115 relieved high fat diet induced obesity. *Lactobacillus paracasei* N1115 decreased body weight, fat accumulation and hyperlipidemia by increasing the abundance of beneficial bacteria (*Allobaculum* and *L. Zeae*) and decreased harmful bacteria (*Turicibacter*).

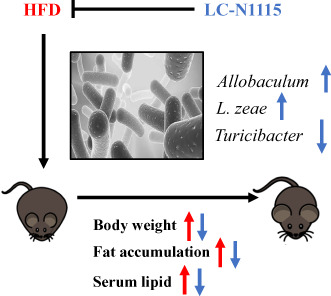

## 1 INTRODUCTION

Currently, most cases of obesity in individuals are caused by a high‐fat diet (HFD) and sedentary lifestyle. Worldwide, the prevalence of obesity has approximately tripled since 1975. Globally, approximately 1.9 billion adults were overweight, and of these, more than 650 million were obese in 2016 (Sarmiento Quintero et al., 2016). Obesity is closely associated with type 2 diabetes, heart disease, and various cancers; including breast, ovary, esophagus, colon, liver, pancreas, endometrium, and prostate cancers; and several other diseases, which not only destroy people's lives but also increase the costs for obese individuals (Mathur & Barlow, 2015). Therefore, health problems such as obesity and related metabolic diseases must be urgently addressed.

Losing weight through drugs or surgery leads to adverse side effects and internal body damage. Alternatively, a restricted calorie diet with physical exercise is the healthiest approach to lose weight. Fatty acids disrupt intestinal barrier function by decreasing the expression and distribution of tight junction proteins (Regnier et al., 2021). Additionally, the gut microbiota composition is altered in an obese individual. Several studies have demonstrated that gut microbiota plays a crucial role in obesity and related metabolic complications and have suggested that obesity strongly correlates with altered gut microbiota (Regnier et al., 2021). Increasing studies suggest that obesity has an inseparable relationship with gut microbiota. Evidence from human clinical trials and animal studies has illustrated that probiotics can inhibit pathogenic bacteria (Roy et al., 2006), enhance barrier function (Guo et al., 2017), and promote homeostasis of the gut microbiota (Regnier et al., 2021). In 2006, Turnbaugh et al. demonstrated that the microbiome of an obese individual has an increased ability to harvest energy from the diet, and this trait is transmissible. Colonization of germ‐free mice with an “obese microbiota” resulted in a significant increase in the total body fat compared with those with a “lean microbiota” (Turnbaugh et al., 2006). Subsequently, Vrieze et al. utilized the therapeutic potential of fecal microbiota transplantation (FMT) and demonstrated that FMT from a lean male to an obese male with metabolic syndrome significantly improved insulin sensitivity and increased microbial diversity (Hartstra et al., 2015; Vrieze et al., 2012). Probiotics can potentially alleviate obesity owing to their capacity to modulate the gut microbiota (Marques et al., 2020).

Probiotic supplementation is used as a gut microbiota‐targeted strategy to combat metabolic disorders (Wang et al., 2015). Various beneficial strains, such as *Bifidobacterium* and *Lactobacillus* spp., can alleviate obesity, hepatic steatosis, and systemic inflammation (Ben Salah et al., 2013; Novotny Núñez et al., 2015; Song et al., 2015; Wang et al., 2015; Yoo et al., 2013). *Lactobacillus paracasei* N1115 (*LC‐*N1115), a novel strain with probiotic properties, is isolated from traditional homemade dairy products in Inner Mongolia, China. As a probiotic strain, *LC*‐N1115 exhibits a high‐level resistance to acidic and bile stresses, and its fermented milk products have been patented (Wang et al., 2014). It shows high similarity to the well‐studied probiotic *L. rhamnosus* GG (LGG) and is considered safe for consumption. Therefore, *LC*‐N1115 has a high commercial value. Although several *Lactobacillus* spp. can reduce obesity, different bacterial strains in the same species or genus may have contrasting responses to the same intervention. Thus, it is essential to investigate the modulatory effect of probiotics on the gut microbiota at the strain level (Wang et al., 2015).

Therefore, the aim of this study was to examine whether administration of *LC*‐N1115 could ameliorate obesity and investigate on its possible mechanisms.

## 2 MATERIALS AND METHODS

### 2.1 Animals, diet, and experimental design

Four‐week‐old male C57BL/6J mice were purchased from Beijing HFK Bioscience Co. Ltd. Animals were housed under conditions of constant temperature and humidity (22 ± 2°C, 55 ± 10%), with a 12 h light/dark cycle. During adaptation (first week), all mice were fed a normal diet (ND). After 1 week of acclimatization, they were randomly categorized into two groups: one group was supplemented with ND as a control (*n* = 8) and the other group was fed HFD (*n* = 32) to induce obesity for 12 weeks (Sun et al., 2020). Furthermore, the diet‐induced obese mice were randomly classified into the following groups (*n* = 8): obese mice receiving HFD and placebo (HFD group); those receiving HFD and a daily high dose of *LC*‐N1115 (NH, 5 × 10^10^); those receiving HFD and a daily moderate dose of *LC*‐N1115 (NM, 5 × 10^8^); and those receiving HFD and a daily low dose of *LC*‐N1115 (NL, 5 × 10^6^) via oral gavage for 12 weeks.

*LC*‐N1115 was isolated from a traditional fermented dairy product of Inner Mongolia, a source of *Lactobacillus* strains with potential probiotic properties. It was grown in MRS broth at 37°C, incubated overnight, and harvested by centrifuging at 3000 *g* and 4°C for 15 min. The probiotic was suspended in a sterilized saline solution and was supplemented to mice via oral gavage.

Body weight was measured once a week. Liver and adipose tissues, such as subcutaneous, epididymis, and mesenteric tissues, were collected from the mice immediately following euthanasia. The tissues were rinsed with phosphate‐buffered saline and weighed. The epididymal fat and liver were frozen in liquid nitrogen for real‐time polymerase chain reaction (PCR) analysis. Stool samples were collected 1 day before sacrifice. Before sacrifice, the mice were fasted for 12 h and anesthetized with diethyl ether.

### 2.2 Blood analysis

Serum samples were obtained by centrifuging the blood samples at 3170 *g* for 15 min to detect total cholesterol (TC), triglyceride (TG), and high‐/low‐density lipoprotein cholesterol (HDL‐c/LDL‐c). These blood indicators were detected using an automatic biochemical analyzer (Hitachi 7600‐020) at the core clinical laboratory of No. 3 Hospital of Beijing University.

### 2.3 Histological analysis of liver and epididymal adipose tissues

Liver and epididymal white adipose tissues were dissected and fixed by immersion in 4% w/v formaldehyde in phosphate buffer at room temperature for 24 h and were embedded in paraffin for staining with hematoxylin and eosin (H&E). A fraction of the main liver lobe was fixed frozen in Tissue‐tek in liquid nitrogen. Frozen sections were sliced and stained with oil red O at 60°C for 10 min using 0.5% oil red O dissolved in propylene glycol. The sliced sections were then counterstained, and images were obtained under a microscope at 200× magnification.

### 2.4 Microbial analysis of the fecal contents

Genomic DNA was extracted from the fecal samples of all mice using the bead‐beating method. The extracted DNA was used as a template for the amplification of the V4 region of the 16S rRNA. Herein, specific primers with barcodes, i.e., primer sets containing Illumina adapters, were used. Subsequently, the DNA samples were sequenced using the Illumina MiSeq platform. Clean reads were clustered into 16S rRNA operational taxonomic units (OTUs) within the threshold of 97% identity using UCLUST. The α‐diversity index was calculated using Mothur. The results of the β‐diversity principal component analysis (PCA) and nonmetric multidimensional scaling (NMDS) were computed using Jaccard.

### 2.5 Real‐time quantitative PCR

Total RNA was extracted from the liver and adipose tissues using TRIzol reagent for quantitative reverse transcriptase PCR analysis. RNA purity was assessed using NanoDrop (Thermo Fisher). Total RNA was reverse transcribed into cDNA using a reverse transcription kit. SYBR Premix Ex Taq, (TaKaRa) was used for RT. All reactions were performed in a single 96‐well reaction plate. Data were calculated using the 2^−ΔΔct^ method, and GAPDH was used to normalize the data. The primers used in this study are listed in Table S1.

### 2.6 Statistical analysis

Data were analyzed using SPSS 21 and were represented as mean ± standard deviations. Statistical comparisons were performed using two‐way analysis of variance followed by Duncan's multiple range tests, and *p* < .05 was considered statistically significant.

## 3 RESULTS

### 3.1 Effect of *LC*‐N1115 treatment on body weight and fat mass of obese mice

**FIGURE 1:**
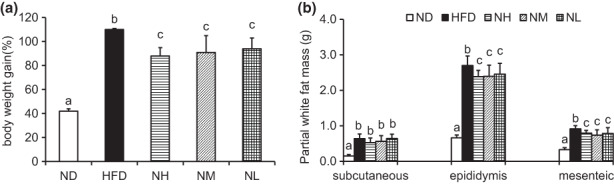
Effects of HFD and probiotic supplementation on body weight (*n* = 8). Effects of probiotic supplementation on (a) body weight gain. (b) Partial white fat mass. Bars not sharing common letter superscripts are significantly different (*p* < .05).

After feeding HFD for 12 weeks, the mice in the HFD group were heavier than those in the ND group by approximately 16% (34.52 ± 1.90 vs. 27.9 ± 1.21 g). The obese mice with HFD were successfully established. After probiotic supplementation for 12 weeks, the body weight of the animals in the NH, NM, and NL groups was lower than those in the HFD group by approximately 9.6%, 12.2%, and 10.1% (39.68 ± 1.77 vs. 43.50 ± 2.30 g, 38.76 ± 2.45 vs. 43.5 ± 2.30 g, and 39.5 ± 1.44 vs. 43.5 ± 2.30 g). These results suggest that *LC*‐N1115 supplementation could counteract the weight gain induced by HFD (Figure 1a).

The HFD group also showed a significant increase in the white fat mass compared with the ND group. Probiotic supplementation decreased fat accumulation as the partial white fat mass of the probiotic group was lower than that of the HFD + placebo group (Figure 1b).

### 3.2 Effect of *LC*‐N1115 treatment on blood lipid level in obese mice

HFD increased the TC, TG, and LDL‐c levels but decreased the HDL‐c levels (Table 1). As shown in Table 1, the plasma TC level of the HFD group was 83% higher than the ND group. The TC levels of mice supplemented with probiotics reduced, particularly in the NL group, by approximately 46.79%. Compared with the ND group, TG levels in the HFD group increased by 54.44%. *LC*‐N1115 treatment decreased the TG levels by 20.14%, 8.63%, and 17.99% in the NH, NM, and NL groups, respectively. LDL‐c levels in the HFD group were significantly elevated compared with those in the ND group. In contrast, the plasma LDL‐c levels of mice supplemented with probiotics were reduced by approximately 9.3%, 20.48%, and 31.59% in the NH, NM, and NL groups, respectively. However, the plasma HDL‐c levels were similar between the HFD and probiotic groups, and plasma TG levels were not significantly different. HDL‐c/LDL‐c, HDL‐c/TC, and TG/HDL‐c ratios indicated the atherogenic indices. The higher the HDL‐c/LDL‐c and HDL‐c/TC ratios, the lower would be the atherosclerosis risk. Furthermore, lower TG/HDL‐c ratio indicated that the individuals were healthy. The HDL‐c/TC ratio in the HFD group was significantly decreased compared with that in the ND group. All doses of probiotic treatment increased the HDL‐c/TC ratio. Furthermore, the HDL‐c/LDL‐c ratio in the ND and HFD groups was 5.14 and 2.62, respectively; however, the HFD group receiving probiotics showed increased ratios of 3.39, 3.23, and 3.51 in the NH, NM, and NL groups, respectively. In addition, the TG/HDL‐c ratio in the probiotic groups was lower than that in the HFD group. In particular, this ratio in the NH group was 0.44, which was 43% lower than that in the HFD group. The other two groups also showed different levels of improvement. In summary, *LC*‐N1115 treatment attenuated hyperlipidemia, but no dose response was observed with the probiotic supplementation.

**TABLE 1:** Effects of LC‐N1115 on serum lipid

	ND	HFD	NH	NM	NL
TG (mM)	0.90 ± 0.04^a^	1.39 ± 0.08^b^	1.11 ± 0.11^c^	1.27 ± 0.05^bc^	1.14 ± 0.06^bc^
TC (mM)	2.64 ± 0.09^a^	5.46 ± 0.17^b^	5.10 ± 0.15^b^	3.35 ± 0.29^c^	2.75 ± 0.27^ac^
LDL‐cholesterol (mM)	0.33 ± 0.01^a^	0.82 ± 0.03^b^	0.76 ± 0.02^b^	0.63 ± 0.03^c^	0.54 ± 0.03^d^
HDL‐cholesterol (mM)	1.73 ± 0.06^a^	2.06 ± 0.04^b^	2.38 ± 0.08^c^	2.11 ± 0.05^b^	2.07 ± 0.06^b^
HDL‐c/TC	0.66 ± 0.01 ^a^	0.39 ± 0.001^b^	0.49 ± 0.02^c^	0.68 ± 0.003^d^	0.64 ± 0.04^a^
HDL‐c/LDL‐c	5.17 ± 0.25^a^	2.68 ± 0.13^b^	3.17 ± 0.39^c^	3.30 ± 0.56^c^	3.40 ± 0.14^c^
TG/HDL‐c	0.44 ± 0.05^a^	0.65 ± 0.08^b^	0.49 ± 0.04^a^	0.59 ± 0.04^b^	0.64 ± 0.06^b^

*Note*: Results are expressed as the means ± SE (*n* ≥ 6). Bars not sharing common letter superscripts are significantly different (*p* < .05).

### 3.3 Effect of *LC*‐N1115 on hepatic and epididymal steatosis

**FIGURE 2:**
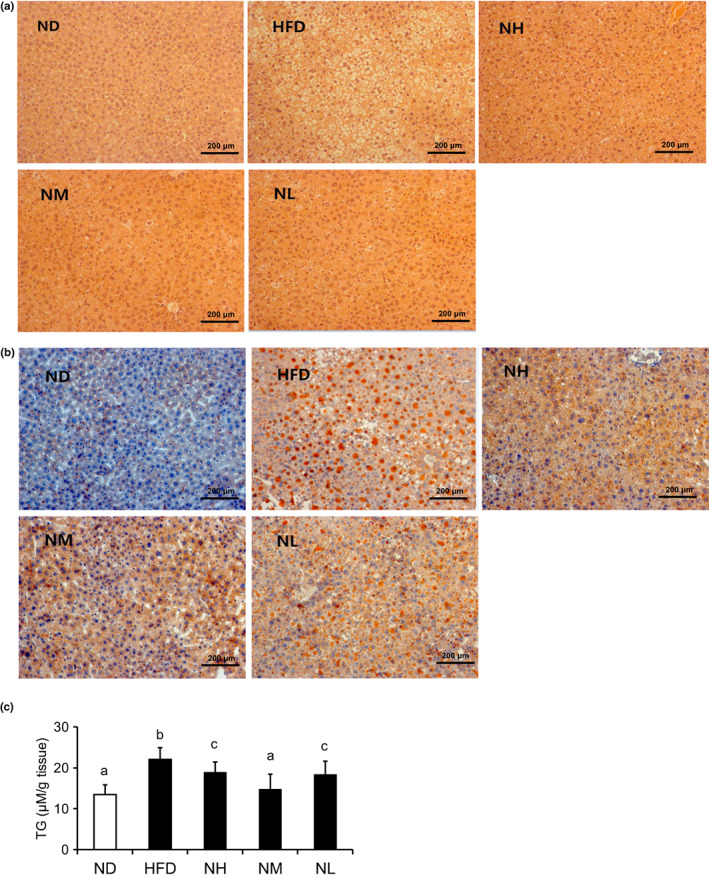
Probiotics attenuated HFD‐induced hepatic steatosis (*n* ≥ 6). (a) Hematoxylin and eosin‐stained liver sections. (b) Oil red O‐stained liver sections. (c) The concentration of TGs in the liver. Bars not sharing common letter superscripts are significantly different (*p* < .05).

We used H&E staining to verify the effect of *LC*‐N1115 on preventing the development of liver steatosis. As shown in Figure 2, oil red O and H&E staining showed fat accumulation in the liver. In Figure 2a, H&E staining of liver sections showed evident ballooning at the hepatocellular level in the HFD group. Compared with the HFD group, the NH, NM, and NL groups almost normalized the liver histology because of the probiotic treatment. Oil red O staining revealed the accumulation of lipid droplets (orange‐red) in the HFD group, and *LC*‐N1115 inhibited lipid droplet deposition in the liver, as shown in Figure 2b. As shown in Figure 2c, TG levels in the liver were measured using enzyme‐linked immunosorbent assay. The results showed that HFD increased the hepatic TG levels, whereas *LC*‐N1115 decreased the stored fat accumulation significantly.

### 3.4 Effect of *LC*‐N1115 treatment on the gut microbiota

**FIGURE 3:**
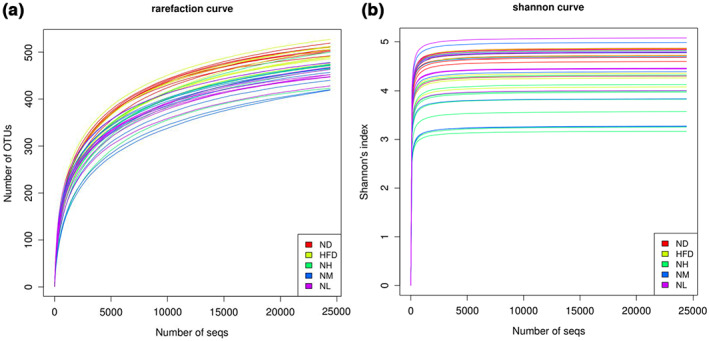
α‐Diversity analysis of the fecal samples. (a) Rarefaction analysis. (b) Shannon diversity index curves

To analyze the effect of probiotics on the microbiota structure, we performed sequencing of the V4 region of the bacterial 16S rRNA gene from the fecal samples. Sequencing reads were assigned to OTUs with 97% sequencing similarity for assessing alpha diversity and were represented using rarefaction curves. At this sequencing depth, the rarefaction curves appeared to plateau in the samples, as shown in Figure 3a. Shannon diversity indices for all samples were stable, as shown in Figure 3b. These results showed that most of the diversity had been covered.

**FIGURE 4:**
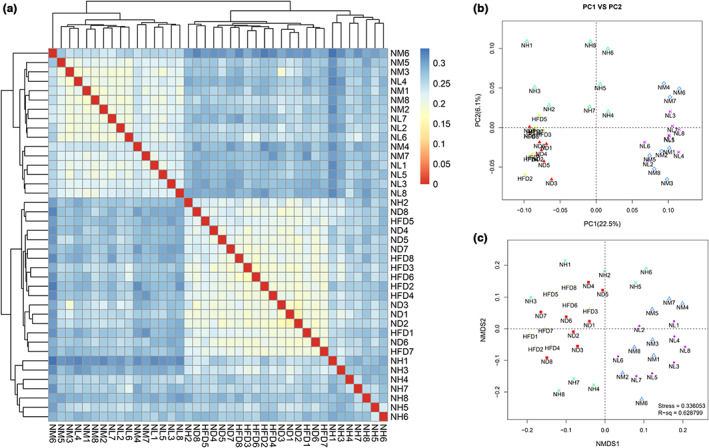
Effect of *LC*‐N1115 on the structure of the gut microbiota. (a) Heatmap based on Jaccard distances. (b) Principal coordinates analysis based on Jaccard distances of OTUs. (c) NMDS based on Jaccard distances of OTUs. The color represents community distances in the heatmap, and the smaller the distance (red), the higher the community similarity.

The differences among the gut microbial communities (β‐diversity) were examined using heatmap, PCA, and NMDS according to the abundance of OTUs, as shown in Figure 4. As shown in Figure 4a, the gut microbial communities in the NM and NL groups showed more similarities. The heatmap and PCA plots could not completely discriminate between the ND and HFD groups, as shown in Figure 4a,b. While the PCA and NMDS plots showed separation between the probiotic and HFD groups (Figure 4b,c).

**FIGURE 5:**
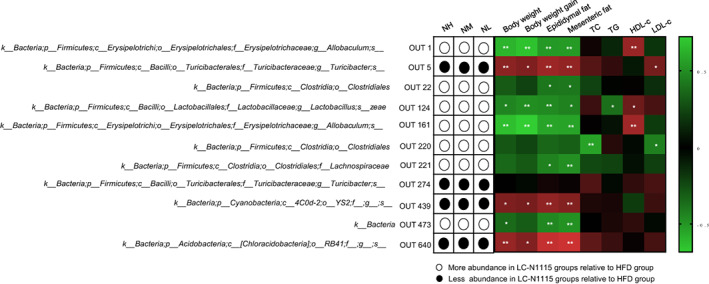
*LC*‐N1115 altered OTUs that were significantly correlated with the host MS parameters (*n* ≥ 7). Asterisk (*) represents OTUs whose abundance was altered by a HFD. However, this change was reversed using three doses of *LC*‐N1115. * represent *p* < .05, * represents *p* < .01

HFD feeding altered the abundance of 264 OTUs. Supplementation with NH, NM, and NL enhanced or reduced the abundance of 131, 104, and 126 OTUs, respectively, resulting in changes in 204 distinct OTUs. Spearman correlation analysis was performed to detect the association between the gut microbiota (changed by *LC*‐N1115) and the obesity index. Significant correlations were obtained between the obesity‐related indices (body weight, white fat mass, TC, TG, and HDL‐c/LDL‐c) and the abundance of the gut microbiota. Among these taxa, *Allobaculum* and *Lactobacillus* were strongly negatively correlated with body weight and white fat mass, suggesting that they play the most crucial role in preventing obesity. In addition, *Turicibacter* was positively correlated with the body weight, white fat mass, and LDL‐c level, indicating that it plays an essential role in the development of obesity. Notably, three doses of *LC*‐N1115 reversed the changes in 11 OTUs that were altered by HFD, as shown in Figure 5. Furthermore, the OTUs that were negatively correlated with the obesity phenotypes were enriched, and all doses of *LC*‐N1115 treatment reduced the OTUs that were positively correlated with the obesity phenotypes. These results suggest that the *LC*‐N1115 treatment altered the gut microbiota of obese mice in both composition and structure.

### 3.5 Effect of *LC*‐N1115 treatment on the proinflammatory cytokine and adipogenesis gene expression in the adipose tissue and liver

**FIGURE 6:**
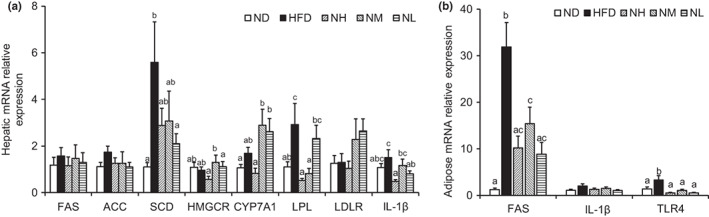
*LC*‐N1115 affects the gene expression in the hepatic and epididymal fat tissues (*n* ≥ 5). Lipid and proinflammatory mRNA expression levels in the (a) hepatic and (b) adipose tissues. Bars not sharing common letter superscripts are significantly different (*p* < .05)

We measured the proinflammatory cytokine and adipogenesis gene expression in the epididymal adipose tissues and liver. As shown in Figure 6a, the expression of the lipolysis gene lipoprotein lipase in the liver was enhanced in the HFD group and significantly reduced in the NH and NM groups. The expression of fat synthesis genes, such as acetyl‐CoA carboxylase, fatty acid synthase (FAS), and stearoyl‐CoA desaturase‐1 (SCD‐1) were enhanced in the HFD group and were found to be reduced in the *LC*‐N1115 group, although not significantly. In the probiotic groups, the expression of the 3‐hydroxy‐3‐methylglutaryl‐CoA reductase (HMGCR) gene was not significantly altered compared with that in the HFD group. The cholesterol 7‐hydroxylase (CYP7A1) and LDL receptor (LDLR) mRNA expression in the NM and NL groups was enhanced compared with that in the HFD group. The expression of the proinflammatory cytokine gene, IL‐1β, was significantly reduced in the NH group and was also reduced in the NM and NL groups but had no significant difference.

In epididymal adipose tissues, as shown in Figure 6b, the FAS gene expression was significantly reduced in the *LC*‐N11115 treatment groups compared with that in the HFD group. The mRNA expression of toll‐like receptor 4 (TLR4) was significantly enhanced in the HFD group compared with that in the ND group and was significantly reduced in mice receiving *LC*‐N1115 treatment. The expression of the IL‐1β gene was reduced in the probiotic groups compared with that in the HFD group; however, the difference was not significant.

## 4 DISCUSSION

Although obesity is not fatal, related metabolic diseases threaten the safety of human life. Structural disorders of the gut microbiota and low‐grade inflammation are significant characteristics of obesity. Studies have reported that feeding HFD to C57BL/6J mice results in obesity. The C57BL/6J mice strain used in this study was highly susceptible to diet‐induced obesity. Abundant scientific evidence supports the role of probiotics in the prevention of obesity. Probiotics have multiple and diverse effects on the host: enhancing barrier integrity, modulating the immune system, and fighting against pathogenic bacteria. Modifying the gut microbiota composition via probiotics is an essential role for modulating energy metabolism, and it acts as a critical modifier of obesity and its related metabolic parameters (Cerdó et al., 2019). In our study, we investigated whether *LC*‐N1115 treatment attenuates obesity in HFD‐induced obese mice and attempted to determine the underlying mechanism.

Obesity is caused when the caloric intake exceeds the energy expenditure, resulting in excess energy. The significant characteristic of obesity is chronic low‐grade inflammation. However, whether systemic inflammation associated with diet‐induced obesity originates from the adipose tissues or intestinal inflammation remains unclear (Moya‐Pérez et al., 2015). HFD disrupts the composition of the gut microbiota, resulting in a decline in the abundance of intestinal bacteria, which are beneficial to the host (*Bifidobacterium*), and an increase in the abundance of pathogenic bacteria (*Desulfovibrionaceae*; Zhang et al., 2010). Although signaling pathways and mechanisms underlying systemic inflammation have not been fully understood, several pieces of evidence have shown that the LPS–TLR4 signaling pathway plays a vital role in systemic inflammation. Recent studies have suggested that LPS can increase intestinal permeability and promote the activation of TLR‐4. Several other studies have shown that cluster of differentiation 14 (CD14) and TLR‐4 signaling mediate the functions of the innate immune cells, which trigger the secretions of proinflammatory cytokines such as IL‐1β along with the activation of the NF‐κB signaling pathway (Zou et al., 2015). Evidence from several studies has shown an association between obesity and chronic systemic inflammation. *LC*‐N1115 showed a downregulatory effect on the mRNA expression of inflammatory cytokines, confirming its weight‐losing function.

Each dose of *LC*‐N1115 induced significant changes in the overall gut microbiota structure that were partially ameliorated by HFD‐induced structural dysbiosis. *Allobaculum* produces short‐chain fatty acids (SCFAs), such as butyrate and propionate (Parada Venegas et al., 2019). SCFAs are well known as essential metabolites that maintain intestinal homeostasis (Parada Venegas et al., 2019). SCFAs can be absorbed by intestinal epithelial cells as fuel and enhance the gut barrier (Parada Venegas et al., 2019). Mendes et al. demonstrated that supplementation with a probiotic mixture (*L. acidophilus*, *L. rhamnosus*, and *Bifidobacterium bifidum*) could regulate the inflammatory responses in mice with colon cancer by altering the microbiota structure. The abundance of *Allobaculum* increased significantly in the probiotic group (Mendes et al., 2018). Zheng et al. suggested that rats with gestational diabetes mellitus supplemented with *Lactobacillus* and *Bifidobacterium* can decrease fasting glucose levels by modulating the gut microbiota. In this study, the abundance of *Allobaculum* was strongly positively correlated with amino acid and bile acid metabolism in rats with gestational diabetes mellitus (Zheng et al., 2022). Spearman correlation analysis revealed that abundance of *Allobaculum* was negatively correlated with body weight and fat mass and positively related with HDL‐c levels. *LC*‐N1115 also increased the abundance of *Zeae*, which belongs to *Lactobacillus*. Zhou et al. suggested that *L. zeae* might have an immunomodulatory function and play a beneficial role in relieving inflammation (Zhou et al., 2018). The abundance of *L. zeae* was negatively correlated with body weight, fat mass, and serum TG levels, and positively correlated with HDL‐c levels. *LC*‐N1115 reduced the abundance of *Turicibacter*, which was identified as potentially harmful bacteria. *Turicibacter* reported a higher relative abundance in patients or mouse models with inflammatory bowel disease, which can induce severe colitis (Bosshard et al., 2002). Evidence showed that in the development of nonalcoholic fatty liver disease (NAFLD), the abundance of pathogenic bacteria *Turicibacter* was increased (Jung et al., 2019; Li et al., 2018). In the obesity model, the abundance of *Turicibacter* increased in the HFD group, which was positively correlated with the LPS levels (Xia et al., 2021). *Turicibacter* was positively correlated with metabolic syndrome phenotypes, such as body weight, fat mass, and LDL‐c levels. Therefore, *LC*‐N1115 significantly increased the abundance of beneficial bacteria (*Allobaculum* and *L. Zeae*) and decreased that of harmful bacteria (*Turicibacter*), possibly contributing to weight loss.

We examined the effect of *LC‐*N1115 on reducing obesity. Our results suggest that *LC‐*N1115 reduced body weight gain and white fat tissues. The plasma cholesterol levels of the NM and NL groups were significantly lower than those of the HFD group. All doses of *LC*‐N1115 can reduce the plasma TC levels, which had a statistical significance in the NM and NL but not in NH groups, compared with the HFD group. We measured the plasma LDL‐c and HDL‐c levels to determine the cholesterols that contribute to the change in TC levels. Our results suggest that different doses of probiotics have a distinct effect on plasma cholesterol levels. Accordingly, several studies reported that *B. bifidum* PTCC1644 increased plasma HDL‐c levels and reduced LDL‐c levels, whereas *L. acidophilus* NS1 reduced plasma LDL‐c levels with no change in HDL‐c levels (Harrington et al., 2004; Song et al., 2015). The distinct effects on plasma TC levels may be attributed to different doses of *LC‐*N1115. The effect of *LC*‐N1115 on TC and LDL‐c are not dose dependent. Low dose of *LC*‐N1115 may be more beneficial to ecological niche of gut microbiota. The serum TG level was not significantly different among the groups of obese mice. These results are similar to those of a previous study that performed *Pediococcus acidilactici* M76 treatment on diet‐induced obese mice (Moon et al., 2014). Furthermore, the HDL‐c/LDL‐c, HDL‐c/TC, and TG/HDL‐c ratios were considered as atherogenic indices. Our results of these indices suggest that *LC*‐N1115 positively reduces the risk factor for cardiovascular disease.

We measured the hepatic steatosis using histological analysis, and the results suggested that *LC‐*N1115 reduced fat accumulation in the liver. The orally supplemented probiotic LGG can attenuate the development of high fructose‐induced NAFLD via altering the composition of the small intestinal microbiota, thereby improving the gut barrier function and reducing the portal LPS levels (Ritze et al., 2014). The liver is a crucial organ for cholesterol homeostasis, and HMGCR and CYP7A1 are involved in cholesterol synthesis, cholesterol uptake, and conversion of cholesterol to bile acids. The mRNA expression of HMGCR decreased and that of CYP7A1 increased, suggesting that a reduction in the liver TC levels in the probiotic groups is caused by the suppression of cholesterol synthesis and increased cholesterol conversion. LDLR is a receptor of LDL‐c; its mRNA expression was increased in the probiotic groups (medium and low doses) compared with the HFD group, which may explain the decrease in LDL‐c levels in both groups.

Studies have suggested that obesity is closely associated with the immune system (Chassaing & Gewirtz, 2014). The immune system includes innate and adaptive mechanisms, and obesity is majorly associated with the adaptive mechanism. TLR‐4 is broadly expressed in the cells of the innate immune system, such as macrophages and epithelial cells. We measured the TLR‐4 mRNA expression in epididymal adipose tissues and observed that the expression was significantly decreased in the probiotic groups compared with that in the HFD group. The activation of adipocytes via TLR‐4 results in the synthesis of proinflammatory factors, such as TNF‐α and IL‐1β (Wolowczuk et al., 2008). The expression of TLR‐4 suggests that *LC‐*N1115 has an antiinflammatory effect. Some studies have reported that probiotic strains can improve the intestinal barrier function, thereby reducing LPS release from the intestinal epithelial cells and leading to decreased proinflammatory cytokine production in the adipose tissues (Cani et al., 2007). These results suggest that *LC*‐N1115 can be beneficial for reducing inflammation; however, its underlying mechanism needs further investigation.

## 5 CONCLUSION

Body weight gain and white fat mass were reduced in HFD‐induced obese mice treated with *LC*‐N1115. The abundance of beneficial and pathogenic bacteria was also regulated in mice receiving *LC*‐N1115 treatment. These findings suggest that *LC*‐N1115 supplementation may provide a natural alternative to attenuate obesity.

## CONFLICT OF INTEREST

The authors declare that they have no conflict of interest.

## ETHICAL APPROVAL

The animal protocol was approved by the Institutional Animal Care and Ethics Committee of the China Agricultural University (Aw80402202‐5‐1).

## Supporting information


Table S1

## Data Availability

The datasets generated during and analyzed during the current study are available from the corresponding author on reasonable request.
