# Supplementary material for: Lactobacillus paracasei N1115 attenuates obesity in high‐fat diet‐induced obese mice
Source: Food Sci Nutr. 2022 Oct 20;11(1):418–27. doi: 10.1002/fsn3.3073 (PMC9834814; doi:10.1002/fsn3.3073)
Supplement: Supplementary file 1 — Table S1 [file FSN3-11-418-s001.docx]

Supplement table 1. Sequences of primer used for RT-PCR

| Gene | Primer sequences |
| --- | --- |
| FAS | Forward:5'-AGACCTGCCGACCACCAT-3' |
|  | Reverse:5'-GCTCCTGCCTCCACTGACT-3' |
| ACC | Forward:5'-TGACAGACTGATCGCAGAGAAAG-3' |
|  | Reverse:5'-TGGAGAGCCCCACACACA-3' |
| SCD | Forward: 5’-CCGGAGACCCCTTAGATCGA-3’ |
|  | Reverse: 5’-TAGCCTGTAAAAGATTTCTGCAAACC-3’ |
| HMGCR | Forward:5'- TGTGGGAACGGTGACACTTA-3’ |
|  | Reverse:5'- CTTCAAATTTTGGGCACTCA-3’ |
| CYP7A1 | Forward:5'- ACAGAAGCATAGACCCAA-3' |
|  | Reverse:5'- TGCCAAACAGCGTTAGAT-3' |
| LPL | Forward:5'-AACAATCTGGGCTATGA-3’ |
|  | Reverse:5'- CCACCTCCGTGTAAATC-3’ |
| LDLR | Forward:5'- AGCAGTGAGTGTATCCATCG-3’ |
|  | Reverse:5'- AATGCAGGAGCCATCTGCAC-3’ |
| IL-1β | Forward:5'-CTTCAGGCAGGCAGTATC-3' |
|  | Reverse:5'-CAGCAGGTTATCATCATCATC-3' |
| TLR4 | Forward:5'-AATCTGGTGGCTGTGGAG-3' |
|  | Reverse:5'-CCCTGAAAGGCTTGGTCT-3' |
| GAPDH | Forward: 5'-TCTCCTGCGACTTCAACA-3' |
|  | Reverse:5'- TGGTCCAGGGTTTCTTACT-3' |
